# Similar Representations of Sequence Knowledge in Young and Older Adults: A Study of Effector Independent Transfer

**DOI:** 10.3389/fpsyg.2016.01125

**Published:** 2016-08-23

**Authors:** Jonathan S. Barnhoorn, Falko R. Döhring, Edwin H. F. Van Asseldonk, Willem B. Verwey

**Affiliations:** ^1^Cognitive Psychology and Ergonomics, MIRA Institute, University of TwenteEnschede, Netherlands; ^2^Sportwissenschaftliches Institut, Universität des SaarlandesSaarbrücken, Germany; ^3^Department of Biomechanical Engineering, MIRA Institute, University of TwenteEnschede, Netherlands

**Keywords:** sequence learning, aging, discrete sequence production task, motor skills, transfer

## Abstract

Older adults show reduced motor performance and changes in motor skill development. To better understand these changes, we studied differences in sequence knowledge representations between young and older adults using a transfer task. Transfer, or the ability to apply motor skills flexibly, is highly relevant in day-to-day motor activity and facilitates generalization of learning to new contexts. By using movement types that are completely unrelated in terms of muscle activation and response location, we focused on transfer facilitated by the early, visuospatial system. We tested 32 right-handed older adults (65–75) and 32 young adults (18–30). During practice of a discrete sequence production task, participants learned two six-element sequences using either unimanual key-presses (KPs) or by moving a lever with lower arm flexion-extension (FE) movements. Each sequence was performed 144 times. They then performed a test phase consisting of familiar and random sequences performed with the type of movements not used during practice. Both age groups displayed transfer from FE to KP movements as indicated by faster performance on the familiar sequences in the test phase. Only young adults transferred their sequence knowledge from KP to FE movements. In both directions, the young showed higher transfer than older adults. These results suggest that the older participants, like the young, represented their sequences in an abstract visuospatial manner. Transfer was asymmetric in both age groups: there was more transfer from FE to KP movements than vice versa. This similar asymmetry is a further indication that the types of representations that older adults develop are comparable to those that young adults develop. We furthermore found that older adults improved less during FE practice, gained less explicit knowledge, displayed a smaller visuospatial working memory capacity and had lower processing speed than young adults. Despite the many differences between young and older adults, the ability to apply sequence knowledge in a flexible way appears to be partly preserved in older adults.

## Introduction

Western societies are aging. This development calls for a better understanding of how age interacts with health and capabilities. Older adults show declining performance in the cognitive and physical domains, resulting in reduced motor performance and changes in motor skill development (Salthouse, 2004; Voelcker-Rehage, 2008). These declines are correlated with reduced neural integrity (Seidler et al., 2015) and associated with more widespread engagement of neural resources, possibly in order to compensate for the reduced integrity (Seidler et al., 2010). Research has indicated that aging may have distinct effects on different aspects of motor learning: complex tasks are affected more than low-complexity tasks and fine motor performance is affected more than gross motor performance (Voelcker-Rehage, 2008). We here focus on changes in motor learning and more specifically, how cognitive representations of motor skills differ between older and young adults. We investigated sequence representations using a discrete sequence-learning paradigm (Verwey, 1999; Abrahamse et al., 2013) in which sequence knowledge was transferred between key-press (KP) and lower arm flexion-extension (FE) movements. A flexible application of motor skills is the basis of day-to-day motor activity and allows generalization of learning to new conditions and contexts.

### Sequence Learning and Transfer of Sequence Knowledge

The ability to apply sequence knowledge in a flexible way is assumed by most models of sequence learning. For instance, the influential scheme of motor learning by Hikosaka et al. (1999) proposes that a sequence is learned simultaneously using two independent systems: an early system based on visuospatial coordinates, and a late system using motor coordinates. The visuospatial coordinate system is more dependent on attentional capacity and is believed to allow for transfer of sequence knowledge to other effectors. A second model of sequence learning that has received much attention is the dual-system theory by Keele et al. (2003). They propose that a multidimensional and a unidimensional system together facilitate learning. The multidimensional system can be implicit or explicit, is protected by attentional constraints and is associated with the ventral pathway. The unidimensional system only facilitates implicit learning, and is associated with the dorsal pathway. In a transfer task, the multidimensional system is thought to enable the use of previously learned stimulus-stimulus associations with a new response mode. A third model of sequence learning is the Cognitive framework for Sequential Motor Behavior (C-SMB) proposed by (Verwey et al., 2015). This framework suggests that, depending on the task, sequence learning can develop at three levels of cognitive processing: the perceptual, central and motor level. This framework also allows for transfer of sequence knowledge, based on associations on the perceptual (e.g., visuospatial) and central (e.g., central-symbolic, potentially using explicit knowledge) level. Unsurprisingly, none of the three models discussed here perfectly accounts for all aspects of motor learning. For instance, Hikosaka et al. (1999) does not specifically address central levels of sequence representation; the model by Keele et al. (2003) is subject to ongoing debate about the definition of a dimension; and the C-SMB model (Verwey et al., 2015) has not been extensively validated on the neural level. However, the models do share the prediction that early in learning, an effector independent representation develops that can be used in situations where novel effectors are used. Furthermore, in all three models, the system facilitating transfer is attention-driven.

In accordance with these models, there is now much evidence that people are able to apply sequence knowledge flexibly when using different types of movements. For example, studies have shown transfer of sequence knowledge from finger-movements to arm-movements (Cohen et al., 1990; Grafton et al., 1998), inter-manual transfer of sequence learning with finger-movements (Verwey and Wright, 2004; Parsons et al., 2005; Verwey and Clegg, 2005; Wiestler et al., 2014), and inter-limb transfer of sequence learning with FE movements with the forearm (Kovacs et al., 2009). A recent review of the transfer literature is provided by Shea et al. (2011). Based on these results, the ability of effector-independent transfer of sequence knowledge in young adults is well supported. However, less is known about how this ability is retained over the life span and whether age affects the representations that are developed.

### Older Adults, Sequence Learning and Transfer

Studies on sequence learning in older adults have indicated a number of differences in learning compared to young adults. Regarding the rate of acquisition, results have been somewhat ambiguous. Namely, in some studies older adults acquired sequence knowledge less quickly than young adults (Curran, 1997; Daselaar et al., 2003; Shea et al., 2006), in other studies at the same rate (Seidler, 2006) and in yet another experiment older adults even showed more skill during acquisition than young adults (Brown et al., 2009). Sequence complexity may play a role here: differences between young and old become more pronounced with increasing sequence complexity, for instance, when the sequences include a second-order predictive structure (Curran, 1997).

Sequence representations and the corresponding movement patterns that older adults develop seem to be less structured. That is, sequences are less efficiently organized in the smaller subsequences (i.e., called motor chunks) typically found in young adults (Shea et al., 2006; Verwey, 2010; Verwey et al., 2011). These reductions in the ability to apply a structure to the sequence have been found to be related to declines in visuospatial working memory (Bo et al., 2009, 2012). Furthermore, the problems in developing an efficient representation may also be related to the idea that older adults remain more reliant on external guidance (Verwey, 2010). In other words, older adults may be learning the general task, but younger adults also learn the sequence. This explains previous findings that older adults improved more slowly than young adults on a repeated sequence, but improved as much over time as young adults in performing random sequences (Shea et al., 2006). Furthermore, older adults’ difficulty in developing and maintaining a sequence representation is also apparent in consolidation, which has been shown to be reduced compared to young adults (Brown et al., 2009). Clearly, a number of previous findings show that older adults have more difficulty developing efficient sequence representations and maintaining them.

Research on transfer of sequence knowledge can help us understand how representations differ between young and older adults. For example, a study by Dickins et al. (2015) showed that older adults were able to transfer sequence knowledge obtained in a sequential finger-thumb opposition task to the non-practiced hand. However, another study suggests otherwise: Panzer et al. (2011) investigated differences in representations of sequence knowledge between age groups using an interlimb practice paradigm. Young and older adults practiced with their right or left arm on day one and with the contralateral limb on day two. The groups performed either the same visuospatial movement sequence or a visuospatially mirrored movement sequence on the second day. Using this paradigm, the authors found that only the young group benefited from this additional practice on day two, and only when sequence presentation was the same on a visuospatial level (non-mirrored). Older adults did not show a clear benefit of the second day of practice in any of the conditions, suggesting that switching effector imposed more problems for them than for younger adults. The differential outcomes between this study and the Dickins et al. (2015) experiment may have to do with task complexity: Panzer et al. (2011) used much longer sequences (16 vs. 4 elements) that were performed using a novel method of responding, which probably took more time getting familiar with than the simple finger-to-thumb opposition task used by Dickins et al. (2015).

Concluding, although results are not fully consistent, sequence representations in older adults may well be different from those in young people: older adults have more difficulty developing and utilizing sequence knowledge with different effectors.

### Current Experiment

In most previous transfer studies with young (e.g., Parsons et al., 2005; Kovacs et al., 2009) as well as with older adults (Panzer et al., 2011; Dickins et al., 2015), transfer was to the mirrored arm or hand. A part of this kind of transfer could potentially depend on motor representations because sequence practice with one effector has been found to have a bilateral effect in the primary motor cortex (Wiestler et al., 2014). Hence, we cannot be entirely sure what type of representation facilitates transfer between mirrored movements. To further disentangle potential age differences in sequence learning, we here focused on transfer purely facilitated by the visuospatial system that is described by most models of sequence learning (Hikosaka et al., 2002; Verwey et al., 2015). While we will mainly refer to this system using the term visuospatial representation, note that types of central or relational coding may be part of this system too (e.g., Verwey et al., 2015). Accordingly, we chose to investigate transfer of sequence knowledge between two frequently used sequencing paradigms, namely sequences of KP and sequences of lower arm (FE) movements. With this paradigm, the effectors involved are highly independent in terms of muscles activated during the movements and in terms of response locations so that any transfer relies on applying visuospatial representations and is independent of the motor representations that may have developed. We made the visual presentation of the tasks equal for both types of movements to facilitate optimal use of visuospatial representations.

Recently, it has been suggested that transfer of sequence knowledge between different contexts involves the adjustment of existing visuospatial sequence representations (Verwey et al., 2016). This adjustment of visuospatial representations may be used also in our task when the movements of a familiar sequence are adjusted to execute different movements. Earlier research showed that in such a situation transfer may be asymmetric. Specifically, it was found that transfer was higher from FE sequences to KP sequences than from KP to FE sequences (Shea and Aranda, 2005, see Dean et al., 2008). This finding is consistent with the notion that executing an aimed movement in a FE sequence involves more feedback processing and attentional demands than executing a key-press movement in a KP sequence (e.g., Cruse et al., 1990; Glencross and Barrett, 1992). This cognitive effort is likely to interfere more with the adjustment of existing visuospatial sequence representations during an FE movement than during a KP movement. Another finding corroborating that transfer involves adjustment of existing representations is that movement sequences in an endoscopic task showed more transfer of practice from a complex environment with precise movements to an easier task environment than vice versa (Verwey et al., 2005). Hence, we expected more transfer from FE sequences to KP sequences than vice versa.

In the current study, older and young participants practiced two six-element sequences with either right-hand KP or with right arm FE movements. During the test phase that followed, they performed random and familiar sequences with the non-practiced movement type (e.g., KP practice was followed by a FE test phase). We hypothesized that the visuospatial system that young adults use (Hikosaka et al., 1999; Keele et al., 2003; Verwey et al., 2015) works in a similar way in older adults. Thus, we expected that both age groups would be able to transfer sequence knowledge between the movement types and would perform familiar sequences in the test phase faster than the random sequences. However, because of indications of reduced processing speed in older adults (e.g., Salthouse, 2000), we expected less transfer in the older group than in the younger group. In anticipation of this result, we explored whether processing speed is associated with transfer between the two sequencing tasks because a higher processing speed may allow for faster adjustment of the available sequence presentations (Verwey et al., 2016). In addition, we measured visual spatial working memory (VSWM) capacity as this capacity has been associated with the rate of learning, probably because a larger working memory allows for easier memorizing of sequential elements (Bo et al., 2009). Finally, we assessed explicit knowledge of the practiced sequences. This variable has often been found to be associated with higher execution rates, especially when sequence execution rate is limited by some other factor (Verwey, 2015). It has been frequently argued that explicit sequence knowledge is not associated with motor, but instead with more abstract central representations like visuospatial representations (e.g., Jeannerod, 1997), so explicit sequence knowledge might be associated with the amount of transfer in the present study too.

## Materials and Methods

### Participants

We recruited older participants (65–75) via advertisements in local newspapers; young participants (18–30) were students participating for course credits. The older adults were only invited for participation when they reported that none of the following applied: severe motor problems; using a wheelchair; limitations in using the fingers or arms; history of neurological problems or stroke; arthritis or rheumatism; color-blindness. Data from 9 older participants was excluded: five stopped participation or were excluded because task performance led to discomfort or pain in the fingers, wrist or arm; one scored below our cut-off of 23 on the Montreal Cognitive Assessment (MOCA, Nasreddine et al., 2005); two others showed extreme error rates in the test phase; one stopped participation because of a lack of motivation. The remaining 32 older adults (20 females) had a mean age of 69.4 ± 2.7, and scored 27.8 ± 1.9 on the MOCA^1^. The 32 young adults (23 females) had a mean age of 22.4 ± 2.7, with a MOCA score of 28.4 ± 1.4. All participants were right-handed as indicated by the Edinburgh handedness inventory (Oldfield, 1971). The ethics committee of the University of Twente approved the study and all participants provided informed consent.

### Apparatus

**FIGURE 1:**
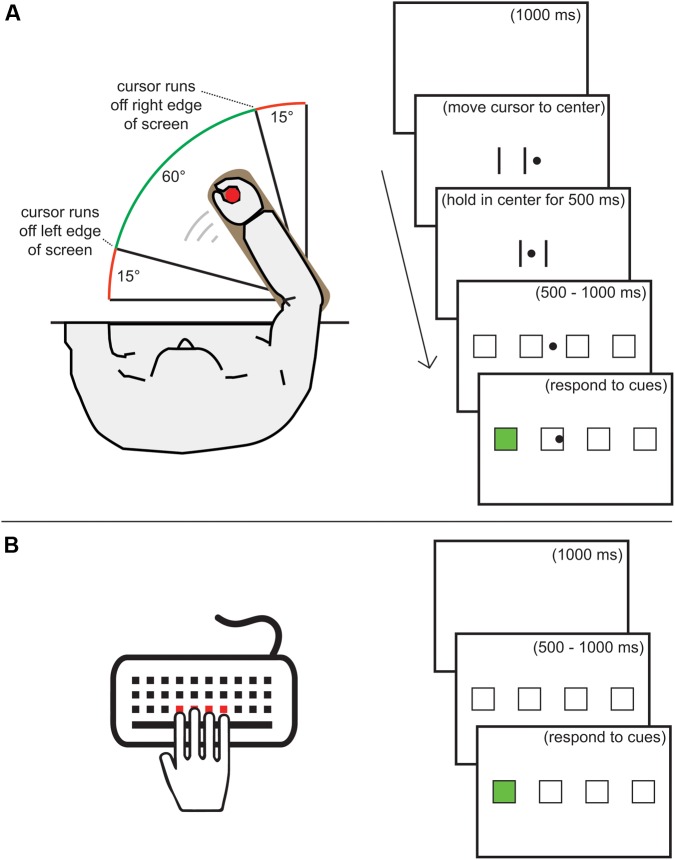
**(A)** Task set-up and visual presentation for the flexion-extension (FE) movements. **(B)** Task set-up and visual presentation for the key-press (KP) movements. The remarks between brackets were not displayed during the tasks.

Participants sat at a table with a 22′ wide-screen monitor placed at 62 cm from the edge of the table. During task performance they responded using either KP movements with the four fingers of the right hand on a standard keyboard or using forearm FE movements with a lever that was fixed to the table (**Figure 1**). The lever was supported by a vertical, nearly friction-less axle. The elbow was aligned with the axis of rotation and the participant held a handle that was shifted according to the length of the arm. An A/D converter attached to a potentiometer recorded the location of the lever at 500 Hz. The FE task was presented using 32-bit Matlab 2014b in combination with PsychToolBox 3.0.11 (Kleiner et al., 2007). The KP and visual spatial working-memory tasks were presented using E-Prime 2.0.

### Design and Procedure

All participants were assigned to one of two task-order groups. They either practiced the sequences using FE movements and were tested on sequence knowledge with KP movements (FE to KP group) or vice versa (KP to FE group).

Participants first completed (1) a handedness form (Oldfield, 1971) at home. The visit to the lab started with administration of the (2) MOCA followed by (3) a VSWM task and (4) a 90 s digit symbol substitution task to assess processing speed (Wechsler, 1955). Then, participants filled in (5) an ad-hoc self-report fatigue scale after which they were given a 3-min break. After this break, the experiment started with (6) a short familiarization phase for each of the movements, starting with the movement type the participant would use during the later test phase. Participants were shown how errors are made and what the error feedback looked like. Then, participants worked through (7) a practice phase using one of the movement types. Following the practice phase, (8) explicit sequence knowledge was measured in three ways using a questionnaire. Participants were first asked to write down the order of the elements using 1 to indicate the leftmost element, 2 to indicate the second, and so on. Then, the target locations were shown on the screen again and participants pointed out the sequences with their index finger. Finally, they were asked to select their sequences from a list of 18 alternatives. After this, (9) a second self-report fatigue scale was filled in and participants started the test phase using the non-practiced type of movements. During (10) the test-phase, a block of random and a block of familiar sequences were performed; the order of these blocks was counterbalanced across participants. After the test phase (11) a final self-report fatigue scale was filled in, participants were debriefed and the experiment was finished. Note that we examined explicit knowledge before the test phase because the order of random and familiar test phase blocks was counterbalanced and the questionnaire would possibly be affected by interference from the random test block in a different way depending on the order.

### Discrete Sequence Production Task

The sequence production task was implemented in a similar way for both types of movements (see **Figure 1**). Black outlines of four 38^∗^38 mm placeholders were horizontally presented with 65 mm spacing between them. A placeholder received a green fill when it became the active target. The screen background was white. When using FE movements, a vertically centered, round, black 8 mm cursor was presented to indicate the location of the lever. Before each trial, this cursor had to be held in the center area of the screen, indicated by two vertical black lines 65 mm apart, for 500 ms. Each trial started with a 500–1000 ms display of the placeholders. When using KP movements, pressing a key during this period resulted in an error message and the display of the placeholders was restarted. When using FE movements and moving the cursor away from the center during this period, the two vertical lines were reinstated and the cursor had to be held in the center for 500 ms again. After the placeholder screen, the first target became active, directly after the correct response the second became active, and so on. When an error was made, the sequence was terminated and a centered red exclamation mark was shown for 500 ms. Between trials, a 1000 ms blank screen was shown.

Before starting the KP familiarization phase, participants were instructed to lay their right-hand fingers on the C, V, B, and N keys and press the spatially corresponding key when a target became active. They also received the instruction that pressing the wrong key or responding too slowly would lead to an error. The maximum RT was 2000 ms when using KP and 3000 ms when using FE movements. Prior to the FE familiarization phase, participants received instruction to use the lever to move the cursor to the active target. Furthermore, the instruction noted that responding too slowly or moving the cursor too far over the target after hitting it (so that the following square or the end of the screen would be hit) would lead to an error. In both familiarization phases, 10 trials of random six-element sequences were practiced. At the start of familiarization and each practice block, participants received the instruction to respond quickly without making too many mistakes (less than 11%).

Before starting the practice phase all participants were told that they were going to learn two sequences of six elements each. Based on our previous research, six element sequences are sufficiently difficult to find individual differences between participants, but easy enough to make sure all participants will learn the sequence to some extent (Abrahamse et al., 2013). They were not told that they would be tested on their sequence knowledge or that they would need to perform a different type of movement later. Practice consisted of six blocks with 48 trials per block and a 120 s break in between blocks. In total, each sequence was performed 144 times. Every block held two sub-blocks with 40 s break in between. During all breaks, the error percentage for the previous sub-block was displayed with a note stating that the participant either made too many errors (when 11% or above), or that he or she did well. Below this, the mean RT in ms was displayed. At the bottom of the screen, a counter showed the remaining time for the break in seconds. The spacebar had to be pressed to start the next block (with the left hand when using FE movements) so that when needed, participants were able to extend the break.

The test phase was performed using the movement type not used during practice. The test phase consisted of one block including the practiced sequences and one block with random sequences. Each test block consisted of 24 trials with a 40 s break in between the blocks. Before starting each test block, participants were informed whether the targets would follow the same order as during practice or no fixed order at all. The order of the two test blocks was counterbalanced over participants.

All participants practiced the same two sequences. The order of elements for sequence A was: 1; 3; 2; 4; 1; 2 (where 1 indicates the left-most target). The order for sequence B was: 4; 1; 3; 2; 4; 3. These sequences are balanced in whether the first location is left or right of the starting position, the total distance covered, and the number of times a one-, two-, or three-element distance is covered. Furthermore, every element is a turning point, making it impossible to hit multiple targets in one sweep, and every target is used three times in total over the two sequences. The sequences were presented in random order. For the random test phase block, the same 24 pseudo-random sequences were used for all participants and presented in random order. For these sequences, all elements were turning points too and locations were never immediately repeated.

### Visuospatial Working-Memory Task

We used a version of the visuospatial working-memory task published by Luck and Vogel (1997, Experiment 1). Each of the 120 trials of the task started with a 1000 ms fixation screen presenting a centered plus sign. Then, participants viewed a sample array of randomly placed colored squares on a gray background for 100 ms. The colors of the squares were randomly determined as well, multiple squares could have the same color. After this, a blank screen was presented for 900 ms, followed by the test array which was presented until the response or until a threshold of 2000 ms had passed. The test array was equal to the sample array except for the fact that one square was encircled. Participants were asked to press “a” when the color of that square was the same or “l” when it was different compared to the sample array, which happened half of the trials. On the top corners of the front of the monitor, reminder labels for the keys were placed: “same” on the left and “different” on the right. After a trial, participants received feedback about whether the response was correct, and could continue to the next trial by pressing “a” or “l.” The array consisted of 2, 4, 6, or 8 squares; every array size was used 30 times. The possible colors were: blue, red, yellow, purple, green, black, and white.

### Analyses

We defined response time (RT) as the time between the onset of an active target and the correct response. Note that after a correct response, the next target became active immediately. For all RT analyses, we excluded the first trial of every sub-block and trials containing an error. Then, we excluded trials with a mean RT that was above a threshold of the mean trial RT plus 2.5 ^∗^ standard deviation of mean trial RTs in that sub-block. Because absolute RTs in older adults and young adults are quite different, we used a percentage transfer score to allow comparison between the age groups. Transfer was calculated as the percentage speed difference between mean RTs of each participant’s familiar and random test block: (random RT – familiar RT)/random RT ^∗^ 100. Note that this score does not control for the amount of learning during the practice phase. For that, a random sequence block would be needed at the end of the practice phase. We decided not to include such a block to prevent potential differential interference effects between the age groups. Learning rate was calculated as the percentage difference between mean RTs of the first and last sub-block of the practice phase: (RT sub-block 1 – RT sub-block 12)/RT sub-block 1 ^∗^ 100. Unless stated otherwise, we report explicit knowledge based on the combined average of the number of elements correctly written down and the number of elements correctly pointed out during the explicit knowledge questionnaire (correct elements were counted from the start to the first mistake). All correlations we report are Pearson product-moment correlations. When the assumptions of sphericity were violated we applied the Greenhouse–Geisser correction, corrected p-values and original degrees of freedom are reported. Proportions of errors were arcsine transformed before analysis (Winer et al., 1991).

## Results

### Practice Phase

**FIGURE 2:**
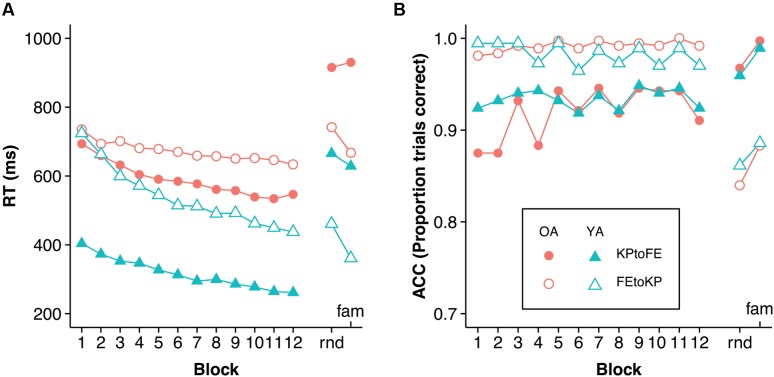
**(A)** RT and **(B)** accuracy development during practice and test blocks. Rnd, random test condition; fam, familiar test condition. The order of test conditions was counterbalanced. Note that regarding FE movements, the older KP to FE group is slower in the test phase than the older FE to KP group is in the first block of the FE to KP condition. Inspection of the FE movements during the familiarization trials suggested a baseline difference between the task groups.

The practice RT and accuracy data were analyzed with a mixed 2 (Age) × 2 (Task) × 12 (Practice Sub-block) repeated-measures ANOVA (see **Figure 2**)^2^. We found an effect of Practice Sub-block, *F*(11,660) = 111.74, *p* < 0.005, $\eta_{p}^{2}$ = 0.651, indicating that participants got faster over time. Older adults were slower than young adults, *F*(1,60) = 68.29, *p* < 0.005, $\eta_{p}^{2}$ = 0.532. The effect of Task was significant too, *F*(1,60) = 38.05, *p* < 0.005, $\eta_{p}^{2}$ = 0.388, indicating that KP movements were performed quicker than FE movements. Age group interacted with Task, *F*(1,60) = 8.15, *p* = 0.006, $\eta_{p}^{2}$ = 0.12, indicating that the RT difference between young and older adults was larger in the KP than in the FE task. Age group interacted with Practice Sub-block too, *F*(11,660) = 8.15, *p* < 0.005, $\eta_{p}^{2}$ = 0.12, suggesting that, overall, young adults improved more than older adults. Practice Sub-block did not interact with Task, *F*(11,660) = 1.44, *p* = 0.226, indicating that when Age group is disregarded, learning rates were not significantly different between the tasks. The three-way Task × Age group × Practice Sub-block interaction, *F*(11,660) = 11.52, *p* < 0.005, $\eta_{p}^{2}$ = 0.161, showed that improvement in the KP task was similar for the Age groups, whereas in the FE task it was lower for the older adults.

Analysis of errors made during the practice phase indicated a main effect of Practice Sub-block, *F*(11,660) = 3.13, *p* < 0.005, $\eta_{p}^{2}$ = 0.05 (see **Figure 2**). Furthermore, the amount of errors differed significantly between tasks, *F*(1,60) = 67.3, *p* < 0.005, $\eta_{p}^{2}$ = 0.529. The interactions with, and main effect of, age group were not significant.

### Test Phase

**FIGURE 3:**
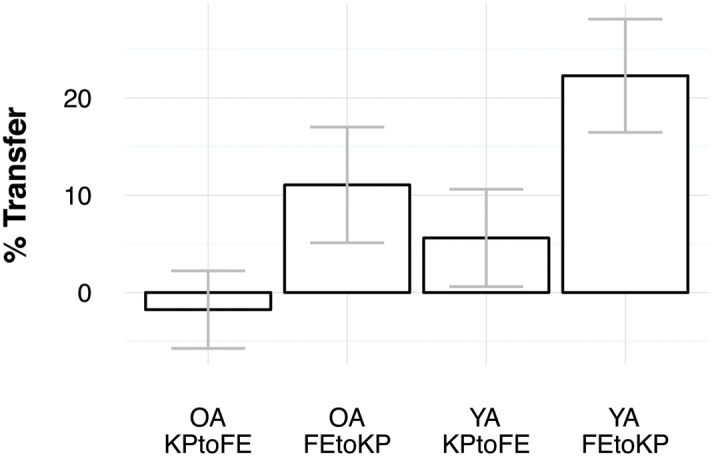
**Percentage transfer for both age groups and task-orders.** Error bars indicate confidence intervals.

To determine whether transfer scores were larger than zero, we performed one-sample *t*-tests on the sub-samples based on task-order and age. Young adults showed transfer in the KP to FE condition [*M* = 5.6%, *t*(15) = 2.39, *p* = 0.031] and in the FE to KP condition [*M* = 22.3%, *t*(15) = 8.17, *p* < 0.005] (see **Figures 2** and **3**). Older adults showed transfer in the FE to KP condition [*M* = 11.1%, *t*(15) = 3.96, *p* < 0.005] but not in the KP to FE condition [*M* = -1.8%, *t*(15) = -0.94, *p* = 0.361]. Group comparison with a 2 (age) × 2 (task-order: FE to KP vs. KP to FE) ANOVA indicates that older adults showed less transfer than young adults, *F*(1,60) = 14.25, *p* < 0.005, $\eta_{p}^{2}$ = 0.19, and that there was more transfer in the FE to KP condition than in the KP to FE condition, *F*(1,60) = 35.90, *p* < 0.005, $\eta_{p}^{2}$ = 0.37. The interaction was not significant, *F*(1,60) = 0.61, *p* = 0.437, $\eta_{p}^{2}$ = 0.01.

Analysis of the errors in the test phases with a mixed 2 (Age) × 2 (Task) × 2 (Test block: familiar vs. random) repeated-measures ANOVA indicated that more errors were made in the random test condition than in the familiar test condition, *F*(1,60) = 15.1, *p* < 0.005, $\eta_{p}^{2}$ = 0.201 (see **Figure 2**). Furthermore, more errors were made in the KP test phase than in the FE test phase, *F*(1,60) = 119.09, *p* < 0.005, $\eta_{p}^{2}$ = 0.665. The interactions and main effect of Age group were not significant.

### Explicit Knowledge, Processing Speed, and Visuospatial Working Memory

**FIGURE 4:**
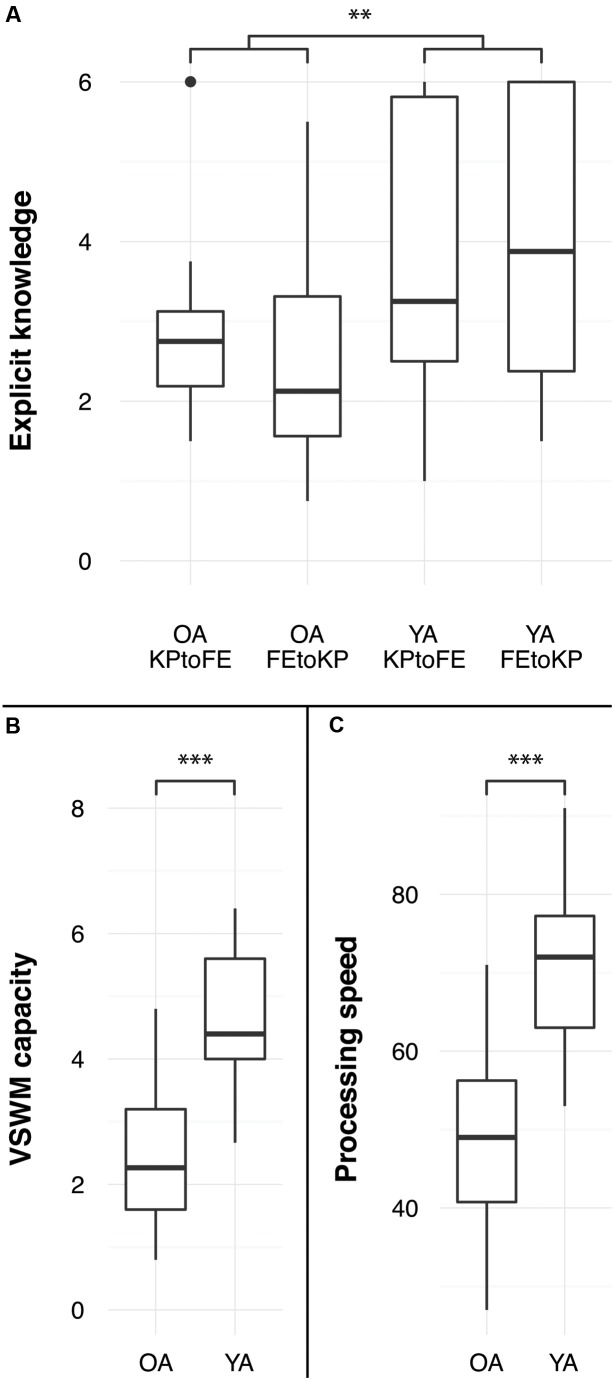
**Age differences in **(A)** explicit knowledge, defined as average number of elements correctly reproduced, **(B)** visuospatial working memory capacity, and **(C)** processing speed.** ^∗∗^*p <* 0.01, ^∗∗∗^*p <* 0.005.

Results from a 2 (age) × 2 (task-order: FE to KP vs. KP to FE) ANOVA on explicit knowledge showed that young adults had more explicit sequence knowledge (3.8 elements per sequence) than older adults (2.6 elements per sequence), *F*(1,60) = 10.58, *p* < 0.005, $\eta_{p}^{2}$ = 0.15. There was no difference between task-order conditions, *F*(1,60) = 0.09, *p* = 0.771, $\eta_{p}^{2}$ = 0.00, indicating that explicit knowledge after the FE and KP practice phases was similar (see **Figure 4**). The interaction effect was not significant, *F*(1,60) = 0.92, *p* = 0.341, $\eta_{p}^{2}$ = 0.02. Explicit knowledge was correlated with transfer in the young adults FE to KP condition but not in any of the other groups (see **Table 1**).

**Table 1:** Correlations between: explicit knowledge and transfer; processing speed and transfer; and visuospatial working memory and learning rate.

	OA, FE to KP	OA, KP to FE	YA, FE to KP	YA, KP to FE
Explicit knowledge ^∗^ Transfer	0.31 (0.12)	0.4 (0.062)	**0.55** (0.014)	0.1 (0.354)
Processing speed ^∗^ Transfer	0.41 (0.056)	0.28 (0.151)	-0.03 (0.548)	-0.14 (0.696)
VSWM ^∗^ learning rate	0.15 (0.284)	**0.49** (0.028)	0.36 (0.086)	**0.5** (0.024)


**P*-values are denoted between brackets, significant correlation coefficients are bold.*

Processing speed was higher in the young than in the old group, *F*(1,62) = 77.60, *p* < 0.005, $\eta_{p}^{2}$ = 0.56 (see **Figure 4**). Correlations between processing speed and transfer were not significant (see **Table 1**).

Young adults had a larger visuospatial working memory capacity than the older adults, *F*(1,62) = 76.15, *p* < 0.005, $\eta_{p}^{2}$ = 0.55 (see **Figure 4**). For older adults, VSWM capacity was correlated with KP learning rate, but not with the FE learning rate (see **Table 1**). We found the same pattern for young adults: VSWM capacity is correlated with KP learning rate, but not with the FE learning rate (see **Table 1**). This VSWM and learning rate relationship for KP sequences is in line with previous research (Bo et al., 2009).

It is important to mention that the small sub-samples (*n* = 16) we used for the correlations together with the added variance from the counterbalanced order of the familiar and random test blocks may have suppressed some effects. Accordingly, inspection of **Table 1** shows that four correlation coefficients were close to significance.

Summarizing, we found that both age groups displayed transfer of sequence knowledge from FE to KP movements, but only young adults showed transfer from KP to FE movements. Older adults showed less transfer than young adults in both tasks. We furthermore found that older adults improved less during FE practice, gained less explicit knowledge, displayed a smaller VSWM capacity and had lower processing speed than young adults. In both age groups, a larger VSWM capacity was associated with quicker sequence learning when performing KP movements but not when performing FE movements.

## Discussion

The models of sequence learning discussed in the introduction (Hikosaka et al., 1999; Keele et al., 2003; Verwey et al., 2015) predict transfer of movement sequence knowledge, even when the actual movements are entirely independent from each other. Our results confirm that both young and older adults showed transfer, although for the older group this was significant only in the FE to KP group and not in the KP to FE group. In line with our predictions, older adults showed less transfer than young adults. Transfer was asymmetric in both age groups: practice with the FE movements followed by a test phase with KP movements resulted in more transfer than vice versa. This is consistent with our expectation that the additional cognitive effort associated with executing FE movements interferes with the adjustment of visuospatial representations, and reduces the ability to use the available sequence knowledge. The observation of correlations between working memory and learning rate in the KP, but not in the FE practice phase, further corroborated that sequence learning in the FE task requires more attention for movement execution and feedback processing than in the KP task. However, note that the correlation between working memory and learning rate in the FE practice phase did approach significance in the young adults. The finding that both age groups showed a similar asymmetry is also a confirmation that the type of representation older adults develops aligns with those that young adults develop. Together, these results suggest that the older participants, like the young, represented their sequences in an abstract visuospatial manner. Since the movement types as well as the response locations were entirely different, we know that the representation used is independent of the reinstatement of a motor representation but instead relies on visuospatial representations (Hikosaka et al., 2002; Verwey et al., 2015). These results suggest that older adults remain able to apply learned motor skills in novel contexts, just like the young.

We found that for both age groups VSWM capacity was correlated with the learning rate when practicing sequences with KP movements. However, when practicing FE movements, both groups showed no relation with VSWM. The KP results are partly in accordance with a study by Bo et al. (2009), who found that visuospatial working memory is correlated with learning rate in young but not in older adults. Why did we find a correlation between learning rate and visuospatial working memory in older adults while Bo et al. (2009) did not? An important reason may be that they used a learning rate score based on the first 60 practice trials of a 12-element sequence while we used more than twice as much practice trials (144) with six-element sequences. In other words, our participants received more practice on an easier task; this may have allowed them to utilize their cognitive capabilities to a larger extent.

We found a correlation between explicit sequence knowledge and the amount of transfer in only one of the four groups. This is unexpected because explicit knowledge has been shown to contribute to sequence production, especially when performing at moderately fast speeds (Verwey, 2015). These results suggest that the early learning mechanism which is usually thought to be processed explicitly (e.g., Hikosaka et al., 2002), also depends, at least partly, on implicit sequence representations. For processing speed, we found only a marginally significant correlation with transfer for the FE to KP older adults group and no other correlations. However, the expectation does hold when comparing the groups: older adults showed a lower processing speed along with less transfer. We expect that the small sub-samples (*n* = 16) we used for the correlations together with the added variance from the counterbalanced order of the familiar and random test blocks may have suppressed some of the correlations. Furthermore, because of the participant’s limited familiarity with the new type of movements when switching to the test phase, factors other than explicit knowledge and processing speed may have played a relatively large role.

In analysis of our results, it is somewhat difficult to control for initial learning because our study did not include a random sequence block at the end of the practice phase because the interference effect may be different over age groups. Future research could contribute to our findings by using either a random sequence block and controlling for interference or by having participants practice until full explicit knowledge of the sequences is reached. The latter option would also allow for more precise inspection of contributions of explicit knowledge. Future research could also consider using a larger sample to gain power when conducting correlational analyses on subgroups.

Concluding, our hypothesis that models of sequence learning that are valid for young adults (Hikosaka et al., 1999; Keele et al., 2003; Verwey et al., 2015) also apply to older adults has been largely confirmed. Older adults learned more slowly, and showed less transfer than young adults when the familiar sequences were carried out with different movements,. This is consistent with earlier indications of reduced transfer (Panzer et al., 2011) and reduced learning (e.g., Curran, 1997; Daselaar et al., 2003) in older adults. However, the types of representations seem to be similar to those that young adults develop and the ability to apply sequence knowledge in a flexible way is partly preserved in older adults.

## Author Contributions

All contributed to conception and design. JB performed data acquisition, analysis, and drafted the manuscript. All contributed to revision and approved the manuscript for publication.

## Conflict of Interest Statement

The authors declare that the research was conducted in the absence of any commercial or financial relationships that could be construed as a potential conflict of interest.

## Footnotes

1 One participant scored 23, and indicated that she was not native Dutch and did not know all the words in the verbal memory task. She fully understood all instructions and was included in the analysis.

2 While we found some indications for heteroscedasticity we report the analyses on the raw RTs. An ANOVA with log-transformed RTs removed the heteroscedasticity but showed the same significance pattern.
